# The role of autophagy in the *Arabidopsis* self-incompatible pollen rejection response

**DOI:** 10.1080/27694127.2022.2065602

**Published:** 2022-04-24

**Authors:** Stuart R. Macgregor, Daphne R. Goring

**Affiliations:** Department of Cell & Systems Biology, University of Toronto, Toronto, Canada M5S 3B2

**Keywords:** ATG5, ATG7, autophagy, plant reproduction, SP11/SCR, self-incompatibility, SRK

## Abstract

In plants, macroautophagy/autophagy is an essential mechanism responsible for a large variety of processes throughout the plant’s lifecycle, including nutrient processing, immunity, stress responses and senescence. Previous studies had observed the presence of autophagosomes in an *Arabidopsis* sexual reproduction system that prevents self-fertilization (self-incompatibility), but their requirement in this pathway was unclear. Using autophagy-deficient mutants, we have recently found that autophagy is a key contributor in the *Arabidopsis* self-incompatibility response to reject self-pollen.

Many flowering plants are hermaphroditic, containing both male pollen-producing anthers and the female pistil, which interact to facilitate sexual reproduction. The mustard family, or Brassicaceae, is a well-studied family which contain both economically important species (such as canola), as well as the model organism *Arabidopsis thaliana*. A number of species in this family have self-incompatibility (SI) systems for the recognition and rejection of SI pollen to prevent inbreeding. For these species, a series of cell-cell communication events occur following pollen delivery onto the stigma, ultimately leading to either the acceptance or rejection of the pollen (Figure 1). When the contacted pollen grain is recognized as SI, it is rejected and remains metabolically inactive ^1^ . In contrast, a pollen grain that is recognized as compatible goes on to germinate and form a pollen tube which will grow through the pistil to an ovule to release sperm cells for fertilization.

**Figure 1.**
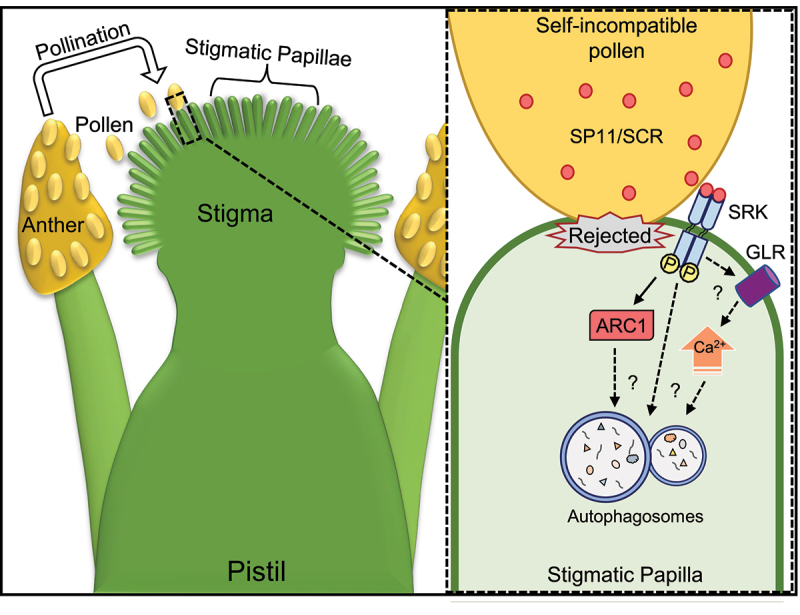
A model for the *Arabidopsis* self-incompatibility pathway. The male pollen is produced in the anther and deposited on the stigma, the receptive end of the female pistil in the flower. Self-incompatible pollen recognition occurs through the binding of a pollen SP11/SCR peptide ligand to the stigma SRK (S receptor kinase). SRK activation results in downstream signaling events which include the potential activation of the ARC1 E3 ligase and glutamate receptor-like (GLR) channels to increase Ca^2+^ levels. Autophagy is activated as part of this cellular response, but how it is activated is unknown. The outcome of this pathway is the rejection of self-pollen by disrupting the basal compatible pollen acceptance pathway.

The Brassicaceae SI pathway is initiated by the interaction between two proteins: the male pollen-derived ligand, SP11/SCR (S locus protein 11) or SCR (S-locus cysteine-rich protein), and the female SRK (S receptor kinase). The multiallelic tandemly-linked genes encoding these polymorphic proteins form a plant’s S-haplotype, and S-haplotype-specific recognition is needed to activate the SI pathway. Activation of SRK triggers the downstream signalling events leading to pollen rejection. While much is known about the SRK-activated pathways in *Brassica* species, less is known for the related *Arabidopsis* species, which appear to employ some different mechanisms for self-pollen rejection. Specifically, autophagosomes were previously found to rapidly appear after SI pollinations in *Arabidopsis lyrata* and transgenic *A. thaliana* plants. Recently, we followed up on this observation to determine if autophagy was essential for the rejection of SI pollen [1].

To investigate the role of autophagy in *Arabidopsis* SI, we crossed the well-established *A. thaliana* autophagy-deficient mutants, *atg7* (autophagy 7) and *atg5*, into the SI lines. The model plant, *A. thaliana*, is a selfing species due to mutations in the SI genes; however, SI can be restored by transforming these genes from related *Arabidopsis* SI species. Two different transgenic *A. thaliana* SI lines were used for this study, and we conducted a detailed examination of the different stages of pollen-pistil interactions. To begin with, pollen hydration was examined as this step is essential for the dehydrated pollen grain to germinate. This step relies on compatible pollen recognition and water release by the stigmatic papilla and is blocked by the SI pathway. Control pollinations of transgenic *A. thaliana* SI pistils with either compatible pollen or SI pollen result in efficient hydration of only compatible pollen as expected. Interestingly, the hydration of SI pollen on the transgenic *A. thaliana* SI pistils is significantly increased when either the *atg7* or *atg5* mutations are present indicating that disruptions in autophagy are breaking down this early stage of SI pollen rejection.

Following pollen hydration and germination, a pollen tube emerges from the compatible pollen grain and enters into the stigma tissue to start growth down towards an ovule, and this is a second step where the SI pathway can block the SI pollen to prevent pollen tube growth. Similar to the pollen hydration step, control pollinations of transgenic *A. thaliana* SI pistils with either compatible pollen or SI pollen result in efficient pollen tube growth through the stigma for only the compatible pollen. When autophagy is disrupted by the presence of the *atg7* or *atg5* mutations in the transgenic *A. thaliana* SI pistils, there is a significant increase in the number of SI pollen tubes growing through the stigmatic tissue, and this results in more SI pollen tubes successfully reaching unfertilized ovules for fertilization. The overall outcome is an increase in the number of seeds produced following SI pollinations when autophagy is disrupted in the transgenic *A. thaliana* SI lines. Thus, the loss of autophagy results in increased inbreeding in these transgenic *A. thaliana* SI lines.

To confirm the rapid appearance of autophagosomes with SI pollinations, direct imaging of autophagosomes was conducted using a GFP-ATG8a fusion protein expressed in the transgenic *A. thaliana* SI pistils. There is a significant increase in the number of visible GFP-labelled puncta in the stigmatic papillae, as early as 10-min post-SI pollination, fitting well with our model of rapidly changing cellular dynamics triggered in the SI pathway. The same increase in the visible GFP-labelled puncta is not observed in the stigmatic papillae when transgenic *A. thaliana* SI pistils expressing GFP-ATG8a are pollinated with compatible pollen.

Despite our study firming placing a role for autophagy in the *Arabidopsis* SI pathway to reject SI pollen, several outstanding questions remain, specifically regarding the mechanisms triggering autophagy and the identity of the cargo in the autophagosomes. While several components involved in SI signalling have been identified, none have been directly linked to the activation of autophagy (Figure 1). Previous studies have implicated an E3-ubiquitin ligase, ARC1 (ARM repeat containing 1), as targeting compatibility factors, including a subunit of the exocyst complex, which is proposed to be involved in vesicle secretion towards the pollen. This is an interesting link given that some exocyst subunits have been connected to both vesicle secretion and autophagic processes. Following these potential connections, the autophagosomes may be carrying cellular components related to secretion for destruction; components that would normally be required for accepting compatible pollen. Previous work has also identified a rapid increase in cytosolic Ca^2+^ levels with SI pollinations, but whether this signal is linked to autophagy is unknown. Last, it remains unclear whether an SI stigmatic papilla is permanently unavailable for additional pollen interactions, once autophagy has been triggered, or if autophagy is used to clear and reset the papilla cell to a basal state, primed to respond to signals from a newly contacted pollen. While our study establishes the requirement of autophagy in SI pollen rejection in *A. thaliana*, the precise nature of its function and the mechanisms which control it will require additional investigation.
